# Efficacy and safety of regorafenib for the treatment of metastatic colorectal cancer in routine clinical practice: results from a Spanish hospital

**DOI:** 10.3389/fonc.2024.1446945

**Published:** 2024-11-12

**Authors:** Pilar Sotoca Rubio, Ana María Barrill Corpa, Víctor Alia Navarro, Patricia Pérez de Aguado Rodríguez, Jaime Moreno Doval, Juan Carlos Calvo Pérez, Patricia Guerrero Serrano, Carlos García Merino, Coral García de Quevedo Suero, Jorge Fernández-Fradejas, Juan José Serrano Domingo, Íñigo Martínez Delfrade, Blanca Isabel Morón García, María Reyes Ferreiro Monteagudo, Belén de Frutos González

**Affiliations:** ^1^ Medical Oncology Department, Ramon y Cajal University Hospital, Madrid, Spain; ^2^ Pharmacy Department, Ramon y Cajal University Hospital, Madrid, Spain

**Keywords:** metastatic colorectal cancer, regorafenib, real-life, routine clinical practice, real world data

## Abstract

**Introduction:**

Regorafenib is indicated as treatment in third-line and beyond in patients with metastatic colorectal cancer.

**Methods:**

This is a retrospective study of a cohort of patients with mCRC treated with regorafenib in Hospital Universitario Ramón y Cajal, in Madrid, Spain.

**Results:**

With the aim to assess the efficacy and safety of regorafenib, 91 patients treated between 2013 and 2023 were included. Only 1.1% of patients achieved disease control. Median progression free survival was 2.40 months and median overall survival was 4.76 months. The most frequent adverse events were fatigue and hand-foot skin reaction (59.34% and 28.57%, respectively).

**Discussion:**

Our results confirm the safety of regorafenib as treatment of mCRC in real clinical practice. Although our population is less pretreated than in the CORRECT trial, our disease control rate was inferior. This difference may be due to a worse baseline status and a high percentage of hepatic disease showed in our patients.

## 1 Introduction

Colorectal cancer (CRC) is the fourth most frequent tumor after breast, prostate, and lung cancers and the third cause of death worldwide. It is estimated that a total of 1,926,425 cases were diagnosed in 2022, with 904,019 deaths. In Europe, 538,584 were diagnosed (1). Approximately 15%–30% of cases are diagnosed as metastatic disease. From those patients diagnosed in localized stages, 20%–50% will have a metastatic relapse. Liver, lungs, and peritoneum are the most frequent areas of metastasis (2).

Several cytotoxic drugs are used in metastatic scenario, with fluoropyrimidines being the most used, in monotherapy or in combination with oxaliplatin and/or irinotecan. These drugs can be combined with monoclonal antibodies, depending on KRAS status. If KRAS is mutated, a drug targeting vascular endothelial growth factor (VEGF) such as bevacizumab can be used, while if it is in a wild-type status, monoclonal antibodies targeting epidermal growth factor receptor (EGFR) like cetuximab or panitumumab can be also used (2). After progression to treatments described above and in patients who maintain good performance status, other options should be offered. Regorafenib or trifluridine/tipiracil are available drugs in this scenario. Trifluridine/tipiracil in monotherapy or in combination with bevacizumab has shown effectiveness and safety in RECOURSE and SUNLIGHT (3, 4).

Regorafenib is a novel oral multikinase inhibitor that blocks VEGF receptor (VEGFR) or EGFR just as additional angiogenic kinases (VEGFR 1/3, platelet-derived growth factor receptor or band fibroblast growth factor receptor 1). Additionally, it blocks regulatory cascades such as RAS-RAF-MEK-ERK and PI3K-PTEN-AKT-mTOR pathways. In summary, regorafenib inhibits tumoral angiogenesis, tumor microenvironment, and cellular signaling (5). The phase 1b clinical trial (BAY 73-4506) showed a tolerable toxicity profile at dose of 160 mg daily for 3 weeks of each 4 weeks and a preliminary evidence of antitumor activity in metastatic CRC (6).

The CORRECT trial was a randomized multicentric phase 3 trial where 760 patients with metastatic CRC were randomized 2:1 to regorafenib or placebo after failure to standard therapies. The primary endpoint was overall survival (OS). Secondary endpoints included progression-free survival (PFS), objective response rate (ORR), disease control rate (DCR), and safety (7). The study met its primary endpoint of OS at the second planned interim analysis, although crossover from the placebo group was allowed after progression. The benefit was modest, with a median OS of 6.4 months in regorafenib group versus 5 months in the placebo group [hazard ratio (HR) 0.77, p=0.0052] (7).

PFS and DCR were also improved with regorafenib. Stable disease was the best response in 41% of patients, and none of the patients reached complete response. In the regorafenib group, 76% of patients had to reduce the dose due to adverse events. The most frequent adverse events of any grade were fatigue and hand–foot skin reaction (especially at first or second cycle). Grade 3 or 4 toxicities appeared in 54% patients in the regorafenib group, mostly hand–foot skin reaction, diarrhea, fatigue, and hypertension (7).

Li et al. were supported in CONCUR, another phase 3 clinical trial with regorafineb in Asiatic population, a similar benefit in OS (8.8 months in regorafenib group vs. 6.3 months in placebo group, HR=0.55, p=0.00016) and a consistent toxicity profile (8).

Based in those clinical trials, regorafenib is nowadays an option for metastatic CRC patients in third line or beyond. Some real-world data (RWD) studies have been published afterwards. Metges et al. confirmed a real-world evidence of efficacy and safety in a prospective and observational study in the 242 French patients who participated in CORRELATE trial. Median OS and PFS were 6.8 months and 2.8 months, respectively (9).

The aim of our study is to evaluate the efficacy and safety of regorafenib in the real-life setting in a tertiary hospital in Spain (Hospital Universitario Ramón y Cajal, Madrid).

## 2 Materials and methods

We conducted a single-center observational, retrospective study of a cohort of 91 patients with metastatic CRC treated with regorafenib in routine clinical practice in third line or beyond between 1 December 2013 and 15 June 2023.

Patients included in the study met the following inclusion criteria: (1) 18 years of age or older; (2) ECOG 0–2; (3) diagnosis of metastatic CRC previously treated with a fluoropyrimidine (alone or in combination with oxaliplatin and/or irinotecan) with or without cetuximab, panitumumab, bevacizumab, or aflibercept; (4) treatment with regorafenib in third line or beyond; and (5) regorafenib as exclusive oncology treatment.

The data of patients were obtained from medical records from the real clinical practice. None of the patients were followed up in a prospective way. Patients were evaluated every 2–3 months with CT scan of the thorax, abdomen, and pelvis according to local guidelines. All re-evaluations were done at our hospital. The main variables assessed were OS, PFS, and ORR according to RECIST 1.1 criteria; adverse events according to NCI-CTCAE (National Cancer Institute Common Terminology Criteria for Adverse Events) versions 4.0 and 5.0; number and dose of cycles of regorafenib and previous; and subsequent treatments received.

The primary objective was to evaluate the effectiveness of regorafenib in patients with metastatic CRC in real clinical practice in terms of PFS. Other objectives were as follows: (1) assess the effectiveness of regorafenib in terms of OS and ORR and (2) know the safety profile in real clinical practice.

Data were analyzed with Stata v16.1. Descriptive statistics were reported as frequencies and percentages for categorical variables and as median and range for continuous variables. Median OS (mOS) and median PFS (mPFS) were estimated using the Kaplan–Meier method and compared by log rank. Cox regression model was used to evaluate the HR and the 95% confidence interval (CI) in univariate analysis.

## 3 Results

A total of 91 patients with metastatic CRC treated with regorafenib in third or beyond line were included. Median age was 65 years (35–85) and 58.24% of the patients (59) were men. Of the patients, 59.34% had metastasis in three or more locations, with 86.81% of them in the liver and 30.77% in the peritoneum. Colorectal cancer had left location in 68.14% of patients. The primary tumor site was the rectum in 24.18% of patients. KRAS/NRAS was mutated in 61.54% of patients. BRAF mutations and MMR deficiency were not found in any patient.

Most patients had ECOG 1 (65.93%), followed by ECOG 0 (20.88%). Only 12.09% of patients had ECOG 2. Regorafenib was administered in third line in 21.98% of patients, and 50.55% of patients had been treated with three or more lines of treatment before regorafenib. In those patients treated in third line, 100% received FOLFOX and FOLFIRI regimens, with 17 (85%) receiving bevacizumab in first and second line and three patients receiving anti-EGFR in first line and bevacizumab in second line.

Regarding trifluridine/tipiracile, 62.64% of patients were treated with this drug before regorafenib and 12.09% of patients received it after regorafenib. A total of 28 patients received other treatments after regorafenib, most of them with 5-fluoruracil.

The most frequent response was progression of the disease, found in 74 patients (81.31%), while 16 patients were not evaluated and only one had disease control.

The characteristics are summarized in **Table 1**.

**Table 1:** Cohort’s characteristics.

Characteristic	Value
Median age, years (range)	65 (35–85)
Sex, no. (%)	
Male	53 (58.24%)
Female	38 (41.76%)
ECOG, no. (%)	
0	19 (20.88%)
1	60 (65.93%)
2	11 (12.09%)
Unknown	1 (1.1%)
Primary location, no. (%)	
Ascendent	27 (29.67%)
Transversum	2 (2.2%)
Sigma	40 (43.96%)
Rectum	22 (24.18%)
KRAS, no. (%)	
Wild type	35 (38.46%)
Mutated	56 (61.54%)
Metastatic affection, no. (%)	
≤2 sites	37 (40.66%)
≥3 sites	54 (59.34%)
Metastatic site, no. (%)	
Liver	79 (86.81%)
Peritoneum	28 (30.77%)
Prior regimens, no. (%)	
2	20 (21.98%)
3	46 (50.55%)
≥4	25 (27.47%)
Best response, no. (%)*	
Stable disease	1 (1.1%)
Progression	74 (81.32%)
Not evaluated	16 (17.58%)
Trifluridine/Tipiracil, no. (%)	
Before regorafenib	57 (62.64%)
After regorafenib	11 (12.09%)
Not received at any time	23 (25.27%)
Subsequent treatment, no. (%)	40 (43.96%)

*According to RECIST 1.1 criteria.

**Figure 1:**
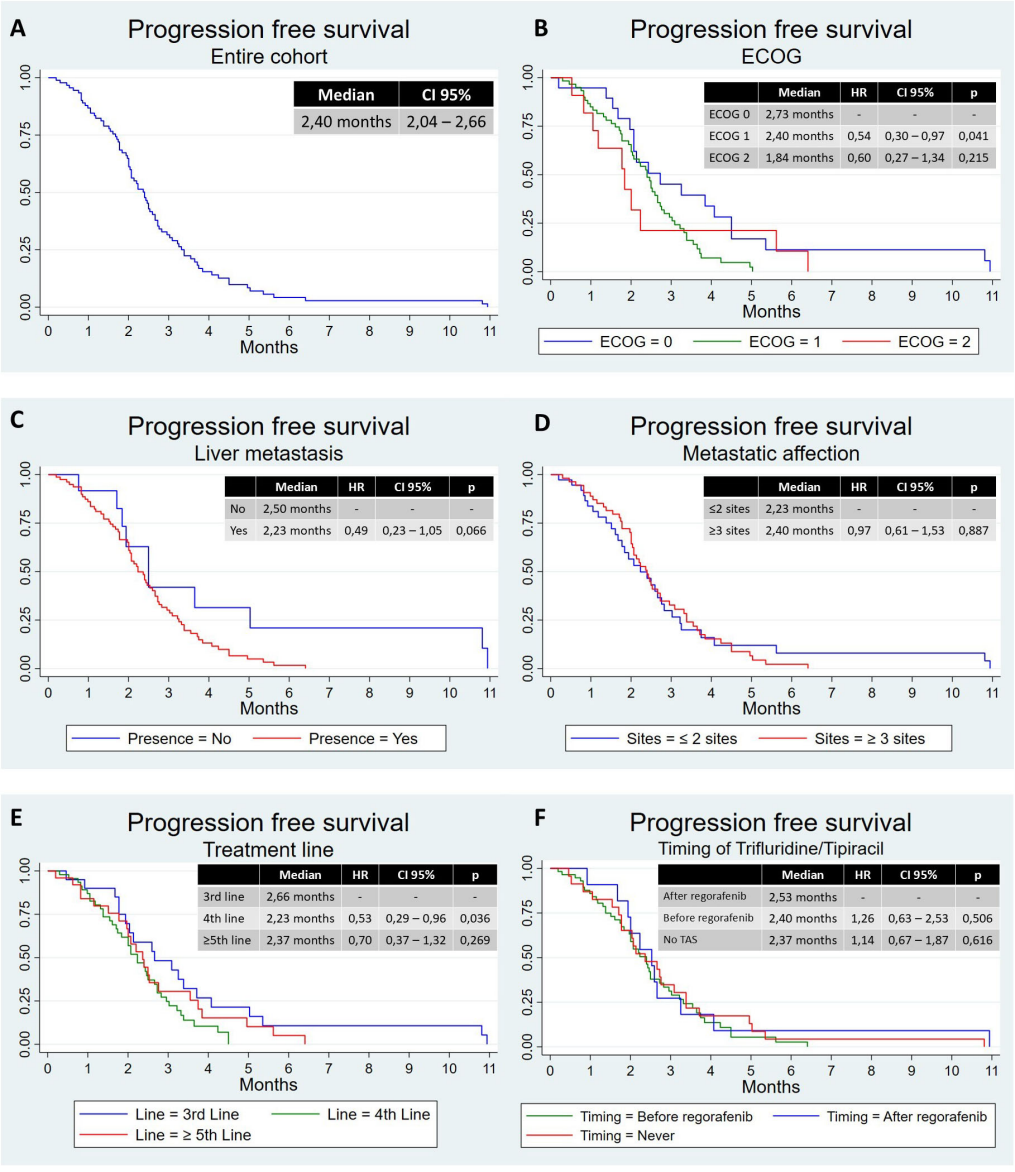
Progression-free survival in different populations. **(A)** Progression-free survival in the entire cohort, **(B)** progression-free survival according to ECOG, **(C)** progression-free survival according to liver metastasis, **(D)** progression-free survival according to metastatic affection, **(E)** progression-free survival according to treatment line, and **(F)** progression-free survival according to timing of trifluridine/tipiracil. CI, confidence interval; HR, hazard ratio; TAS, trifluridine/tipiracil.

**Figure 2:**
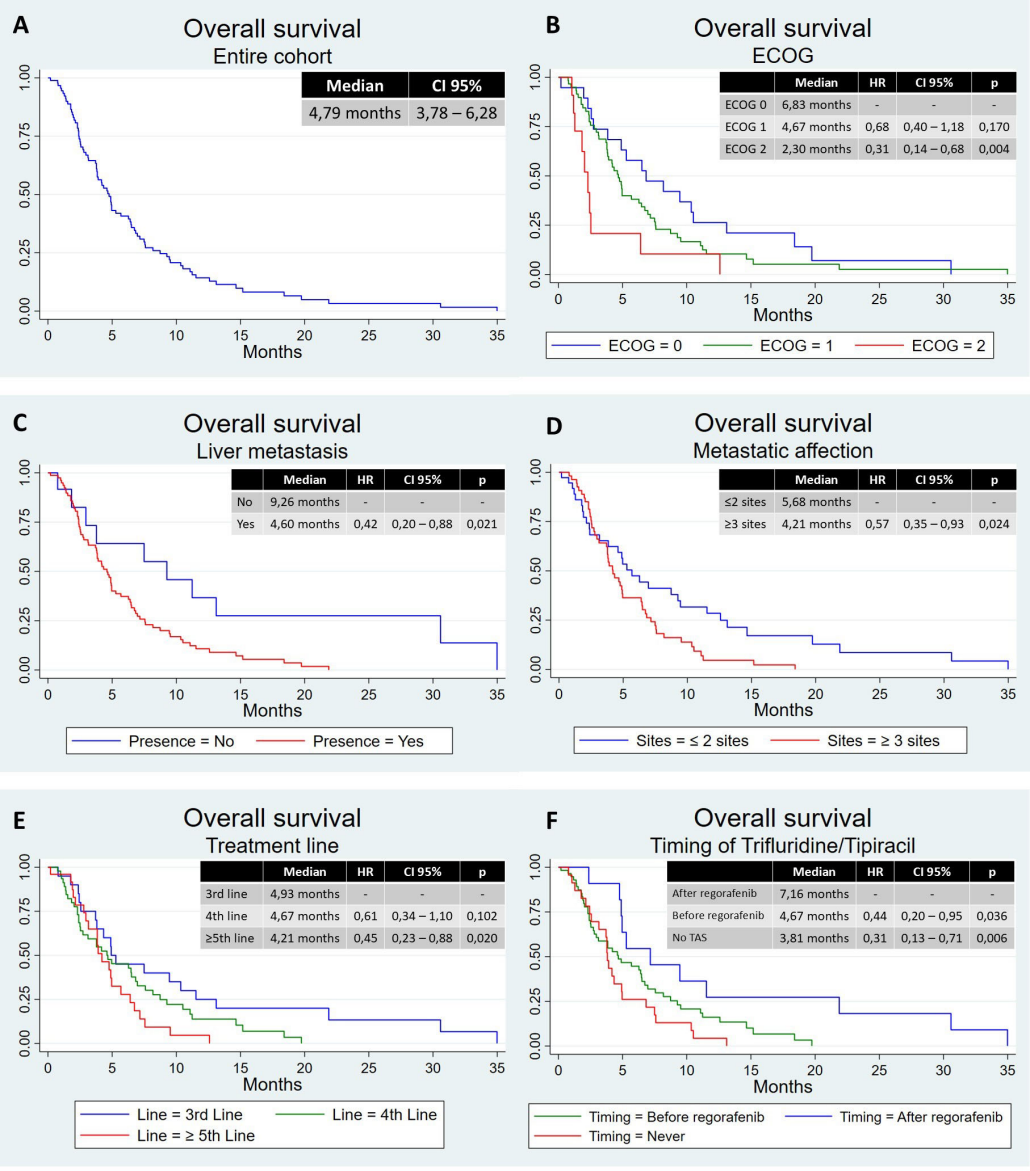
Overall survival in different populations. **(A)** Overall survival in the entire cohort, **(B)** overall survival according to ECOG, **(C)** overall survival according to liver metastasis, **(D)** overall survival according to metastatic affection, **(E)** overall survival according to treatment line, and **(F)** overall survival according to timing of trifluridine/tipiracil. CI, confidence interval; HR, hazard ratio; TAS, trifluridine/tipiracil.

Median follow-up was 6.13 months (range, 0.20–35.0 months). The mPFS was 2.40 months (95% CI, 2.04–2.66 months) (**Figure 1A**), and the mOS was 4.76 months (95% CI, 3.78–6.28 months) (**Figure 2A**). Median duration of treatment was 2.52 months (range, 0.20–10.9 months). According to ECOG, patients with ECOG 0, 1, and 2 or more had an mPFS of 2.73, 2.40, and 1.84 months, respectively. There was a significant difference between ECOG 0 and 1 with an HR of 0.54 (95% CI, 0.30–0.97, p=0.041) (**Figure 1B**). The mOS in these patients was 6.83 months, 4.67 months, and 2.30 months, respectively. ECOG 2 had a worse OS than ECOG 0 (HR 0.31, 95% CI, 0.14–0.68, p=0.004) and ECOG 1 (HR 0.45, 95% CI, 0.23–0.90, p=0.024), with no differences between ECOG 0 and 1 (**Figure 2B**).

Those patients with liver metastasis had worse mPFS, with 2.23 months vs. 2.50 months, not statistically significant (HR 0.49, 95% CI, 0.23–1.05, p=0.066) (**Figure 1C**), and worse mOS, with 4.60 months vs. 9.26 months (HR 0.42, 95% CI, 0.20–0.88, p=0.021) (**Figure 2C**). Patients with peritoneal involvement had similar mPFS (2.37 months vs. 2.40 months; HR 1.03, 95% CI, 0.63–1.67, p=0.909) and similar mOS (4.34 vs. 4.90 months; HR 1.30, 95% CI, 0.80–2.12, p=0.291).

There was no difference between patients with two or less and three or more metastatic sites in terms of mPFS: 2.23 months and 2.40 months (**Figure 1D**). In terms of mOS, there was a difference, with 5.68 months vs. 4.21 months (HR 0.57, 95% CI, 0.35–0.93, p=0.024) (**Figure 2D**). There was no difference in terms of PFS between patients with tumor in the rectum and other locations (mPFS 2.2 months vs. 2.40 months; HR 1.67, 95% CI, 0.99–2.82, p=0.055), neither in OS (mOS 3.78 months vs. 4.90 months; HR, 1.39; 95% CI, 0.83–2.32; p=0.208).

According to treatment line, those patients treated with two prior regimens had better mPFS than those treated with three (2.66 months vs. 2.23 months; HR 0.53, 95% CI, 0.29–0.96, p=0.036). Patients treated with four or more prior regimens had an mPFS of 2.37 months without differences comparing the other groups (**Figure 1E**). In terms of OS, we have observed a better mOS in patients treated with two prior regimens than those treated with four or more (4.93 months vs. 4.21 months; HR 0.45, 95% CI, 0.23–0.88, p=0.020). Patients treated with three or more prior regimens had an mOS of 4.67 months without differences comparing the other groups (**Figure 2E**).

Finally, and considering the sequence with trifluridine/tipiracil, there was no difference in mPFS, with 2.40 months for those treated first with trifluridine/tipiracil, 2.53 months for those treated first with regorafenib, and 2.37 for those not treated with trifluridine/tipiracil (**Figure 1F**). However, those patients treated first with regorafenib had better mOS (7.16 months) than those treated first with trifluridine/tipiracil (4.67 months; HR 0.44, 95% CI, 0.20–0.95, p=0.036) and those not treated with trifluridine/tipiracil (3.81 months; HR 0.31, 95% CI, 0.13–0.71, p=0.006) (**Figure 2F**).

A total of 40 patients (43.96%) started regorafenib at 160 mg/day and 46 (50.55%) with 120 mg/day (one level dose reduction). A total of 26 patients (28.57%) required a dose reduction, without impact in mOS. Regarding safety, 62 patients (68.13%) presented toxicity grades 1 and 2, and 14 patients (15.38%) presented toxicity grades 3 and 4. The safety profile is summarized in **Table 2**.

**Table 2:** Safety profile.

Characteristic	Value
Starting dose, no. (%)	
160 mg	40 (43.96%)
120 mg	46 (50.55%)
80 mg	5 (5.49%)
Dose reductions, no. (%)	26 (28.57%)
Toxicity, no. (%)*	
No toxicity	15 (16.48%)
Grade 1–2	62 (68.13%)
Grade 3–4	14 (15.38%)
Fatigue	
Grade 1–2	53 (58.24%)
Grade 3–4	1 (1.10%)
Hand–foot skin reaction	
Grade 1–2	24 (26.37%)
Grade 3–4	2 (2.20%)
Diarrhea	
Grade 1–2	6 (6.59%)
Grade 3–4	1 (1.10%)
Hypertension	
Grade 1–2	6 (6.59%)
Grade 3–4	3 (3.30%)

mg, milligrams.

*According to National Cancer Institute Common Terminology Criteria for Adverse Events (NCI-CTCAE) versions 4.0 and 5.0.

## 4 Discussion

Our study has assessed the efficacy and safety of regorafenib in third line and beyond in the treatment of patients with metastatic CRC in real clinical practice. The main clinical outcomes included a DCR of 1.1%, an mPFS of 2.40 months and an mOS of 4.76 months. Hand–foot skin reaction was the most frequent adverse event. ECOG 2, liver metastasis, and having three or more metastatic sites showed worse OS, as expected. In a similar way, patients treated in third line have also a better OS than those treated in fifth or greater line. Conversely, the presence of peritoneal disease does not negatively impact survival. Since colon and rectal cancer are actually diseases with biological, clinical, and molecular characteristics (10), we have assessed the impact that this could have in our study. However, no differences in survival had been observed between patients with colon and rectal cancer. This observation is in line with what was observed by the authors of CORRELATE study in relation to the sidedness of the primary tumor (11), reinforcing the idea that prognostic differences are lost in latter lines.

Our results are slightly lower to those observed in the randomized phase III CORRECT clinical trial in terms of OS, with a difference in mOS of 4.76 months vs. 6.4 months. This can also be observed in comparison to the CORRELATE study (7.7 months). This difference may be due to the fact that our population has a worse baseline status. In the CORRECT trial and in the CORRELATE study, 52% and 41% of patients were ECOG 0, respectively, while in our database, it was 20.88%; 12.09% of the patients in our database were ECOG 2, while in CORRELATE, it was 6% and was an exclusion criterion from the CORRECT trial. In our population, 86.81% had liver metastasis, which is recognized as a poor prognostic factor. These data were missing from the CORRECT trial, while it was 72% in the CORRELATE study.

In the CORRECT trial, 27% of patients have received one to two prior regimens, 25% have received three prior regimens, and 49% have received four or more prior regimens. In our database, this corresponds to 21.98%, 50.55%, and 27.47%, which shows that our population is less pretreated than in the CORRECT trial. This may explain that in our study, the mPFS is slightly superior: 2.4 months vs. 1.9 months. In the CORRELATE study, patients treated in the third, fourth, or fifth line and beyond were 30%, 30%, and 39%, respectively, with an mPFS of 2.9 months. The DCR has been clearly inferior in our study, with 1.1% (with only one patient with stable disease) versus 41% in the CORRECT trial and 26% in the CORRELATE study. Different times of revaluation between clinical practice and clinical trial could explain this fact. Patients are usually evaluated every 3–4 months in real clinical practice, while in the CORRECT trial, they were evaluated every 8 weeks. These data were not shown in the CORRELATE study. The median duration of treatment was similar in our study and in CORRELATE (2.5 months), both superior to that in CORRECT (1.7 months). This difference could be also explained because of the different times of revaluation.

Several studies have evaluated the role of the sequence between regorafenib and trifluridine/tipiracil, with better result in OS with the sequence trifluridine/tipiracil followed by regorafenib (12, 13). In contrast, in our study, we have observed contrary results. We suggest that it may be due to the fact that treatment selection is made according to the patient’s characteristics. In general, trifluridine/tipiracil is better tolerated than regorafenib, so those patients with a better performance status could be selected to start regorafenib, given that subsequent clinical deterioration may not allow its use. However, since only 11 patients received the sequence of regorafenib followed by trifluridine/tipiracil, no firm conclusions can be drawn. Furthermore, this question would currently be outdated following the data from the SUNLIGHT study, which showed that the addition of bevacizumab to trifluridine/tipiracil is superior to trifluridine/tipiracil as monotherapy (4).

The safety profile observed in our study had some differences with that in the CORRECT trial and with those reported in other retrospective studies of RWD (11). In the CORRECT trial, 38% of the patients in the regorafenib group needed a dose reduction, similar to the CORRELATE study, where 40% required a dose reduction (11). In contrast, in our study, dose reduction was lesser (28.57%), maybe because 54.94% of patients started at a lower dose. Regarding the CORRELATE study, 43% of patients started at a lower dose, mainly due to their fragility (11).

Hand–foot skin reaction and fatigue were the most frequent adverse events shown in the CORRECT trial, in the CORRELATE study, and in our study, with some differences. In the CORRECT trial, 47% of patients had any grade of fatigue and 47% had any grade of hand–foot skin reaction; in the CORRELATE study, 41% and 26%, respectively (11); and in our study, 59.34% and 28.57%, respectively. We have summarized the differences between the studies in **Table 3**.

**Table 3:** Results comparison.

Outcome	CORRECT^	CORRELATE	Actual series^^
Median age, years (range)	61 (64–67)	65 (24–93)	65 (35–85)
Male, %	62%	61%	58%
ECOG			
0	52%	41%	21%
1	48%	46%	66%
≥2	NA*	6%	12%
KRAS			
Wild type	41%	37%	38%
Mutated	54%	56%	62%
Unknown	5%	7%	0%
Liver metastasis	NS	72%	87%
Peritoneum metastasis	NS	13%	31%
Prior regimens			
1–2	27%	30%	22%
3	25%	30%	51%
≥4	49%	39%	27%
Response evaluation			
Partial response	1%	4%	0%
Stable disease	40%	22%	1%
Progression	59%**	47%	81%
Not evaluated		27%	18%
mTD	1.7 months	2.5 months	2.52 months
mPFS	1.9 months	2.9 months	2.40 months
mOS	6.4 months	7.7 months	4.76 months
Safety			
Dose reduction	38%	40%	29%
Inferior starting dose	NA	43%	56%
Toxicity			
Grade 1–2	39%	45%	68%
Grade 3–4	54%	35%	15%
Fatigue	47%	41%	59%
Hand–foot skin reaction	47%	26%	29%
Diarrhea	34%	19%	8%

NA, not applicable; NS, not specified; mTD, median treatment duration; mPFS, median progression-free survival; mOS, median overall survival.

^These data are from the cohort treated with regorafenib, not the entire population.

^^Data may not be the same as those reflected in this study because percentages have been rounded in this table.

*ECOG 2 was an exclusion criterion.

**Separate data not provided.

It must be taken into account that this is a retrospective study, with the usual limitations of these types of studies. The information collected is limited to that obtained from the patients’ medical records, so it is subject to possible bias and lack of information. In addition, the absence of centralized revaluation could influence the reliability of data in PFS. This is why it is not possible to draw more robust conclusions.

### 4.1 Conclusion

Our results confirmed the efficacy and safety of regorafenib as treatment of metastatic CRC in real clinical practice. There are several differences between the results of clinical trials and studies based on routine clinical practice, such as previous lines administered and rates of control disease. Fatigue and hand–foot skin reaction were the most adverse event associated with regorafenib.

Prospective studies are needed to evaluate which sequence of treatments may be most beneficial for patients with advanced colorectal cancer in later lines.

## Data Availability

The raw data supporting the conclusions of this article will be made available by the authors, without undue reservation.
